# A Functional Approach Reveals a Genetic and Physical Interaction between Ribonucleotide Reductase and CHK1 in Mammalian Cells

**DOI:** 10.1371/journal.pone.0111714

**Published:** 2014-11-06

**Authors:** Lorena Taricani, Frances Shanahan, Maria-Christina Malinao, Maribel Beaumont, David Parry

**Affiliations:** Merck Research Laboratories, Palo Alto, California, United States of America; University of Oxford, United Kingdom

## Abstract

Ribonucleotide reductase (RNR) enzyme is composed of the homodimeric RRM1 and RRM2 subunits, which together form a heterotetramic active enzyme that catalyzes the de novo reduction of ribonucleotides to generate deoxyribonucleotides (dNTPs), which are required for DNA replication and DNA repair processes. In this study, we show that ablation of RRM1 and RRM2 by siRNA induces G1/S phase arrest, phosphorylation of Chk1 on Ser345 and phosphorylation of γ-H2AX on S139. Combinatorial ablation of RRM1 or RRM2 and Chk1 causes a dramatic accumulation of γ-H2AX, a marker of double-strand DNA breaks, suggesting that activation of Chk1 in this context is essential for suppression of DNA damage. Significantly, we demonstrate for the first time that Chk1 and RNR subunits co-immunoprecipitate from native cell extracts. These functional genomic studies suggest that RNR is a critical mediator of replication checkpoint activation.

## Introduction

Ribonucleotide reductase (RNR) catalyzes the reduction of ribonucleotides to deoxyribonucleotides, the essential precursors of DNA synthesis in all organisms. RNR is an important enzyme in the early stages of DNA synthesis responsible for maintaining a balanced supply of dNTPs required for DNA synthesis and repair. Thus, RNR plays an important role in genetic fidelity AND cell viability [1], [2]. Failure to control the dNTP levels leads to cell death and genetic abnormalities [3], [4].

The classical Ribonucleotide reductase of the *de novo* pathway consists of two subunits, RRM1 and RRM2 [5]. The large subunit RRM1 contains the catalytic site, the substrate-specifity site, and the activity site [2]. The RRM2 subunit contains an iron center generated tyrosyl free radical that can be scavenged by hydroxyurea [6]. An additional RRM2 subunit, p53R2 was identified in 2000 [7]. Like RRM2, p53R2 can substitute for RRM2 to form an active enzyme with RRM1 [8].

The key role of RNR in DNA synthesis and cell growth has made it an important target for anticancer therapy [9]–[11]. Non-selective inhibitors of RNR activity such as hydroxyurea (HU), cytarabine (ara-C), clofarabine (CAFdA), gemcitabine (GEM), Trimidox, and Didox have been investigated for the treatment of a wide variety of solid tumors and hematologic malignancies [12]. Many of these antimetabolites suppress dNTP levels and inhibit DNA replication [13]–[15]. Thus, exposure to antimetabolites induces a coordinated series of intra-S checkpoint events that support replication fork stabilization and prevent irreversible fork collapse [16]. According to current understanding, the kinases ATR and Chk1 play critical roles in this checkpoint [17]–[19].

Chk1 is a key downstream effector kinase in cell cycle checkpoint control that becomes activated in response to DNA damage or stalled replication in higher eukaryotes, thus promoting genomic integrity [17], [20]–[24]. Chk1 activity is essential for stabilization of stalled replication forks [17], [19]. Chk1 is also essential for normal development and DNA synthesis [23]–[25]. Despite numerous studies, it remains unclear how replication stress signals induced following exposure to RNR inhibitors are transduced to the appropriate checkpoints and Chk1. Specifically, the interplay between RNR activity, dNTP levels, and the signaling mechanisms that activate Chk1 to ensure appropriate coordination of DNA replication and checkpoint function remain obscure in mammalian cells.

To identify novel genetic interactions with Chk1, we employed an RNAi-based synthetic lethal screen. In this study, we identified gene products that when ablated lead to activation of Chk1 and subsequent synergy in combination with Chk1 siRNA using γ-H2AX, a marker of double-strand DNA breaks as a read out of mechanism [17], [26]. We identified DNA polymerase alpha (Polα) [27] and RNR as strong genetic interactors from this screen. Combinatorial ablation of DNA Polα and Chk1 causes an accumulation of γ-H2AX, suggesting that activation of Chk1 in this context is essential for suppression of DNA damage [27]. Co-depletion of RNR with Chk1 yields similar phenotypes to Polα/Chk1, suggesting that RNR is required for maintenance of genomic integrity following replication stress. Here, we present evidence that RNR is a critical mediator of replication checkpoint activation. We also demonstrate for the first time that analogous to Chk1 and Polα, Chk1 and RNR co-immunoprecipitate *in vivo*. These findings suggest that the Chk1/RNR replication complex is a key component of the replication checkpoint.

## Materials and Methods

### Cell lines, Drugs, and siRNA Treatment

Human U20S osteosarcoma cells, obtained from American Type Culture Collection (ATCC) were grown in DMEM (Mediatech) supplemented with 10% FBS (JRH BioSciences), 200 U/ml Penicillin, 200 µg/ml Streptomycin, and 300 µg/ml L-Glutamine (Cambrex). The siRNA duplexes were purchased from Dharmacon. The siRNA sense sequences used were:

Control siRNA (siLuciferase): CAUUCUAUCCUCUAGAGGAUGdTdT

siChk1: GAAGCAGUCGCAGUGAAGAdTdT

siChk2: CUCUUACAUUGCAUACAUAUU

siRRM1#1: GCACAGAAAUAGUGGAGUAUU*

siRRM1#2: GAACACACAUACGACUUUAUU

siRRM1#3: GGACUGGUCUUUGAUGUGUUU

siRRM1#4: UGAAACGAGUGGAGACUAAUU

siRRM2#1: GCACUCUAAUGAAGCAAUAUU

siRRM1#2: GAACCCAUUUGACUUUAUGUU

siRRM1#3: GAAGAGAGUAGGCGAGUAUUU

siRRM1#5: GAGUAGAGAACCCAUUUGAUU*

Cells were transfected with 50 nM siRNA for Chk1, 100nM siRNA for Luciferase (LUC), CHK2, RRM1, and RRM2 duplexes using Lipofectamine 2000 (Invitrogen) according to the manufacturer's protocol. Asterisk denotes RRM1 #1 and RRM2 #5 siRNA used for experiments.

### Clonogenicity Assay

For clonogenicity assay, following 30 h after siRNA transfections and cells transfected with Luciferase were treated with 1 mM HU for 8 h were re-plated with fresh media at 10,000 cells/well in triplicate wells in 6-well plates and allowed to proliferate for 7 days. Attached cells were fixed with methanol/acetone (1∶1) for 20 min and stained with crystal violet solution (0.2% crystal violet (w/v), 2% ethanol) for 20 min and washed with water.

### Flow Cytometric Analysis

γ-H2AX detection for DNA damage and BrdU incorporation for cell cycle analysis were performed as described previously [17], [27] and analyzed with BD LSR II (BD BioSciences) using FacsDIVA software.

### Apoptosis Measurement

The measurement of cell death was performed using BioCarta reagents (BioCarta) as described previously [17] and analyzed with BD LSR II (BD BioSciences) using FacsDIVA software.

### Western Blot Analysis of siRNA Knockdowns

Cell pellets were trypsinized, washed with PBS, and lysed in 2X SDS sample buffer (Invitrogen). Protein extracts were separated by SDS-polyacrylamide gel electrophoresis and transferred to Immobilon-P membrane (Millipore). Antibodies used in this study were from Santa Cruz (RRM1  = R1, RRM2  = R2, Polα  =  PolA), Cell Signaling (pS345-Chk1, pT68-Chk2, tubulin), Stressgen (Chk1), Bethyl Laboratories (pS33-RPA 32), Neomarker (PRKDC) Oncogene (α-tubulin), BD Biosciences (KSP) and in-house and Chk1 (MAb58D7) [27].

### Immunoprecipitation

Preparation of cell pellets, determination of protein concentrations and CHK1 and Polα immunoprecipitation were all performed as previously described [27].

### NTP/dNTP Extraction

NTP/dNTP extraction and was performed as previously described [28], [29]. All experiments were done in triplicate.

#### Instrument and Chromatographic Conditions and Measurement of NTP/dNTP Levels

Instrument and chromatographic conditions and measurement of NTP/dNTP levels were performed as previously described [29]. Chromatograms are analyzed for peak integration on Unicorn version 5.2 (GE Healthcare) to calculate the area under the curve to quantitate the levels of dNTPs (dCTP, dGTP, dATP, dTTP) and for the NTPs (CTP, GTP, ATP, UTP) in U20S cells.

## Results

### Inhibition of RRM1 and RRM2 deplete dNTP pools, suppress DNA synthesis and induce markers of DNA damage in S phase

**Figure 1:**
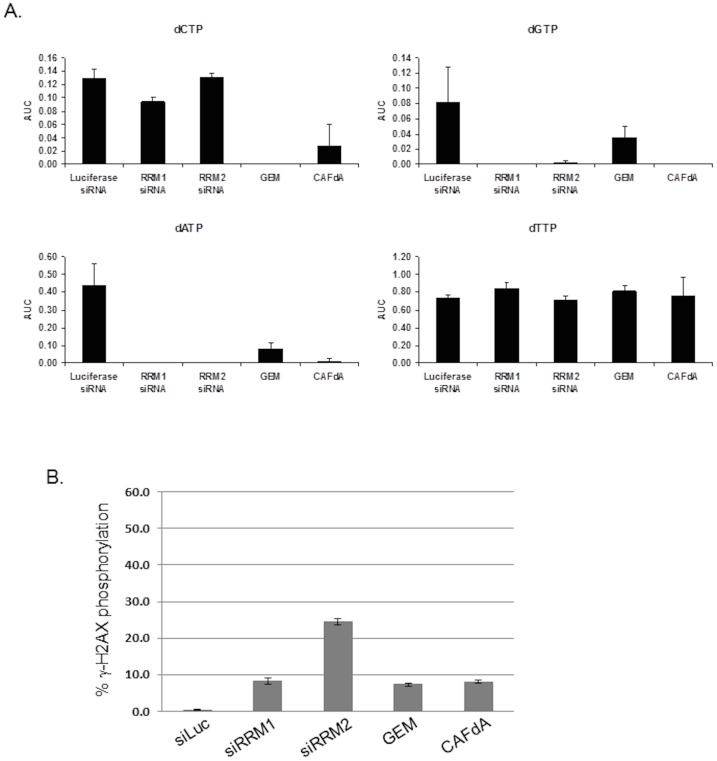
Quantitation of dNTPs and γ-H2AX phosphorylation in U20S cells following depletion of RRM1 and RRM2 subunits of Ribonucleotide reductase. Cells were transfected with RRM1, RRM2 and Luciferase control (untreated or treated with 1 µM GEM or 1 µM CAFdA for the last 2 h) before harvesting at 30 h. At 30 h after siRNA transfections, (a) NTP/dNTP extractions were prepared and quantified. (b) DNA damage was assessed for γ-H2AX phosphorylation using flow cytometry. Data performed in triplicates. Error bars represent standard deviations (SD) between experiments.

Ribonucleotide reductase is a target of hydroxyurea (HU), gemcitabine (GEM), and clofarabine (CAFdA). HU, GEM, and CAFdA are RNR inhibitors, which deplete dNTP pools to inhibit DNA replication [30]–[34]. Subsequent stalled replication or DNA damage induces phosphorylation of Chk1 S345 and γ-H2AX S139 in S phase checkpoint [18], [20], [35]. To evaluate effects on dNTP pools, we compared the depletion of the two RNR subunits, RRM1 and RRM2 with GEM and CAFdA, known inhibitors of RNR. Studies have shown that GEM and CAFdA treatment suppress dCTP and dGTP & dATP pools but do not affect the dTTP pools [32]–[34]. In control cells, transfected with siRNAs directed against luciferase, dGTP was the smallest dNTP pool (0.08±0.05), compared with dCTP (0.13±0.01), dATP (0.43±0.13), dTTP (0.73±0.04) (Figure 1A; Table 1). Ablation of RRM1 or treatment of cells with GEM and CAFdA, known inhibitors of RNR resulted in depletion of dATP, dCTP, dGTP pools but did not affect the dTTP pools (Figure 1A; Table 1). Ablation of RRM2 produced similar outcomes, with the exception of dCTP levels, which remained unchanged when compared to control cells. Thus, specific depletion of RRM1 and RRM2 leads to diminished dNTP pools.

**Table 1:** Quantitation (AUC) of dNTP & NTP pools in the U20S cells.

dNTPs & NTPs	Luciferase siRNA	RRM1 siRNA	RRM2 siRNA	GEM	CAFdA
dCTP	0.13±0.01	0.09±0.01	0.13±0.01	nd	0.04±0.03
dGTP	0.08±0.05	nd	0.0048±0.0	0.03±0.02	nd
dATP	0.43±0.13	nd	nd	0.08±0.04	0.0308±0.0
dTTP	0.73±0.04	0.84±0.08	0.71±0.06	0.81±0.06	0.76±0.21
CTP	5.14±0.36	3.89±0.2	4.30±0.1	4.04±0.06	6.04±0.22
GTP	7.83±0.61	3.88±0.11	4.74±0.08	8.7±0.15	7.68±0.21
ATP	62.42±2.82	42.14±1.4	46.97±1.16	60.41±0.45	52.87±2.67
UTP	14.62±1.18	8.44±0.33	9.38±0.17	15.37±0.39	17.19±0.4

Analysis was done in triplicate.

± denotes standard deviation.

nd  =  not detected.

Units of measurement are AUC (Area under the curve).

Next, we examined if depletion of the RRM1 and RRM2 lead to similar induction of γ-H2AX as RNR inhibitors. siRNA-mediated knockdown of RRM1 (8.1%) for 30 h showed similar levels of γ-H2AX phosphorylation to GEM (7.2%) and CAFdA (8.0%) treatment (Figure 1B). Whereas, knockdown of RRM2 (24.5%) for 30 h shows an increased level of γ-H2AX phosphorylation compared to RRM1 under similar conditions (Figure 1B). The differences in γ-H2AX phosphorylation levels between siRNA-mediated knockdown of RRM1 and RRM2, prompted us to test multiple siRNAs for each gene in U2OS for further confirmation that the effect was specific and not an off-target effect of one a single sRNA. RRM1 and RRM2 expression was ablated using 4 independent siRNAs for each gene for 24 h and 48 h in U2OS cells (Figure S1). All the siRNA duplexes could efficiently knockdown RRM1 and RRM2, which in turned resulted in a G1/S block similar to HU treatment (Figure S1B). Consistently, all siRNA-mediated knockdown of RRM2 exhibited elevated phosphorylation of Chk1 S345 and γ-H2AX S139 compared to RRM1 siRNAs (Figure S1). These data suggest that the effects observed resulting from these treatment with these siRNAs are likely on-target, and equivalent in their activity. Thus, siRRM1#1 and siRRM2#5 were then taken forward in all experiments.

**Figure 2:**
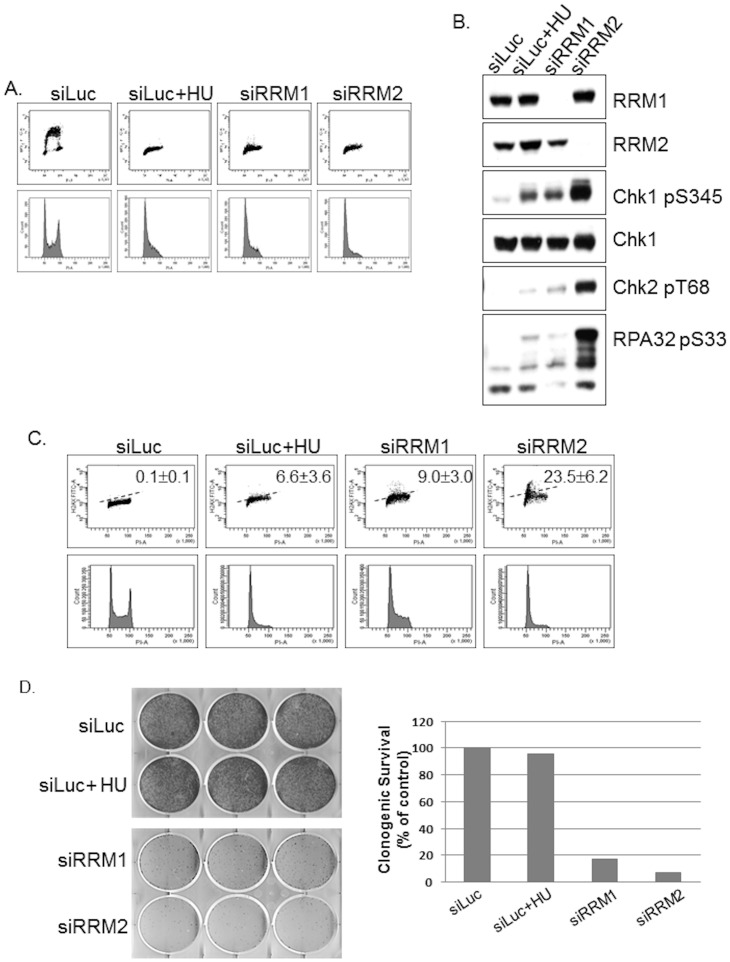
Depletion of RRM1 and RRM2 subunits of Ribonucleotide reductase inhibits DNA synthesis and induces phosphorylation of Chk1 and γ-H2AX in U2OS cells. (a) Following 30 h siRNA transfections, cells transfected with RRM1, RRM2 and Luciferase control (untreated or treated with 1 mM HU for the last 8 h) were stained with BrdU and PI and examined by flow cytometry. (b) Extracts were immunoblotted as indicated. (c) DNA damage was assessed for γ-H2AX phosphorylation using flow cytometry. ± denotes SD. (d) Cell proliferation was assessed with clonogenicity assay. Cell proliferation was expressed as a percentage of luciferase control cells. Results are expressed as the percentage of colony-forming efficiency and are the means of at least three independent experiments.

Since the proposed mechanism of action for hydroxyurea is inhibition of DNA synthesis through RNR inhibition, we hypothesized that ablation of RRM1 and RRM2, the two subunits of Ribonucleotide reductase would result in suppression of DNA synthesis. U2OS cells depleted for RRM1 and RRM2 from asynchronous populations were pulse-labeled with Bromodeoxyuridine (BrdU) and examined by flow cytometry (Figure 2A; Table 2). Table 2 shows the percentage of cells that are able to undergo DNA synthesis and the overall cell cycle distribution, as determined by flow cytometry. These experiments recapitulated the deficiency in G1 to S transition observed in the γ-H2AX experiment (Figure 2C). RRM1 and RRM2 deprived cells accumulated during G1 phase, in a manner that was similar to HU treated cells (76% G1 Luciferase +HU versus 74% and 84.7% G1 for RRM1 and RRM2, respectively at 30 hours). Of note, comparatively fewer cells were detected in S-phase (0.1% S Luciferase +HU versus 1.5% and 0.5% S for RRM1 and RRM2, respectively at 30 hours). Luciferase siRNA transfected control cells were able to incorporate BrdU (24.7% G1 and 55% S Luciferase) and progress through the cell cycle. Thus, RRM1 and RRM2 depletion broadly phenocopies HU exposure; cells lacking these subunits exhibit diminished incorporation of BrdU, suggestive of defects in DNA replication.

**Table 2:** Percentage of cells in G1, S, or G2 phase.

siRNA	%G1	%S	%G2
Luc	24.7	55.0	19.2
Luc +HU	76.0	0.1	8.0
RRM1	74.0	1.5	8.7
RRM2	84.7	0.5	5.4
Chk1	37.3	45.3	15.9
RRM1/Chk1	70.3	1.7	9.3
RRM2/Chk1	88.2	0.5	5.0
Luc + SCH900776 (MK8776)	20.4	57.7	19.2
RRM1 + SCH900776 (MK8776)	71.0	0.8	12.5
RRM2 + SCH900776 (MK8776)	85.7	0.4	6.6

Next, we depleted the two RNR subunits, RRM1 and RRM2 and examined the phenotypic effects by Western blot. Specific depletion of RRM1 phenocopies hydroxyurea exposure and induces readily-detectable phosphorylation of Chk1 S345, Chk2 T68, and RPA32 S33 (Figure 2B). Interestingly, depletion of RRM2 induces significantly more phosphorylation of Chk1 S345, Chk2 T68, and RPA32 S33 compared to depletion of RRM1. To verify that RNR activity directly affects cell proliferation and viability, cells were re-plated following 30 h siRNA-mediated knockdowns of RRM1 and RRM2 subunit of RNR or following 8 hour HU treatment with siRNA-mediated knockdown of Luc, as a positive control. Cells were allowed to grow for a further seven days. Figure 2D shows the growth-inhibitory effects following depletion of RRM1 and RRM2 subunits of RNR. RNR inactivation results in a decrease in dNTPs (Figure 1; Table 1), an inhibition of DNA synthesis and DNA repair, cell cycle arrest and then cell death [36], [37]. Consistent with these observations, depletion of RRM1 or RRM2 results in an accumulation of double-stranded DNA breaks (Figure 2C; Table 3), as measured by flow cytometry using γ-H2AX staining [38], [39]. Ablation of RRM1 and RRM2 for 30 h led to γ-H2AX induction of 9.0% and 23.5%, respectively. Thus, under these conditions, specific depletion of RRM1 or RRM2 induces Chk1 S345, Chk2 T68, RPA32 S33 and γ-H2AX S139 phosphorylation signals correlated with the inhibition of DNA synthesis and stalling of replication forks.

**Table 3:** % H2AX phosphorylation.

siRNA	% H2AX phosphorylation
Luc	0.1±0.1
Luc +HU	6.6±3.6
RRM1	9.0±3.0
RRM2	23.5±6.2
Chk1	19.3±5.7
Chk1 +HU	60.0±3.8
RRM1/Chk1	34.2±3.2
RRM2/Chk1	81.5±2.3
Chk2	0.2±0.1
Chk2 +HU	9.1±1.2
Chk1/Chk2	3.7±0.4
Chk1/Chk2+HU	36.1±1.7
RRM1/Chk2	6.8±0.9
RRM2/Chk2	32.7±1.7
Luc + SCH900776 (MK8776)	25.7±1.8
RRM1 + SCH900776 (MK8776)	84.8±1.5
RRM2 + SCH900776 (MK8776)	94.1±0.6

Data represents 2-independent experiments performed in triplicates.

± denotes standard deviation.

### Identification of RRM1 and RRM2 depletion is synthetically lethal with Chk1 inhibition

**Figure 3:**
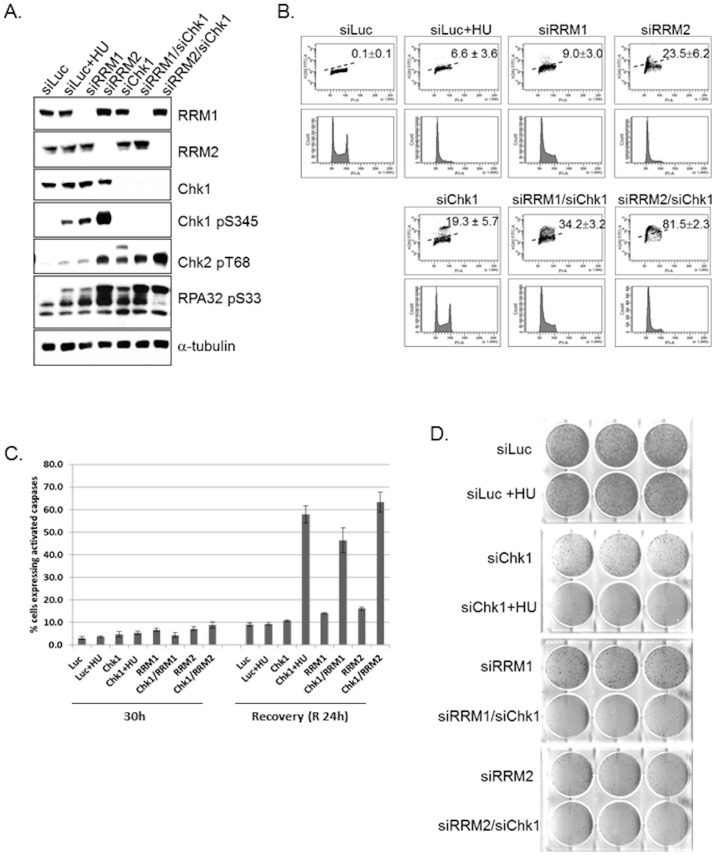
RRM1/Chk1 and RRM2/Chk1 co-depletion enhances the accumulation of DNA damage, apoptotic response and effects cell proliferation in U2OS cells. Cells were transfected with Chk1, RRM1, RRM2, RRM1/Chk1, RRM2/Chk1, and Luciferase control (untreated or treated with 1 mM HU for the last 8 h) before harvesting at 30 h. At 30 h after siRNA transfections, (a) extracts were prepared and immunoblotted with the indicated antibodies. (b) DNA damage was assessed for γ-H2AX phosphorylation using flow cytometry. (c) Cells were collected at indicated time points indicated and analyzed for activated caspases. Data performed in duplicates. Error bars represent SD between experiments. (d) Cell proliferation was assessed with clonogenicity assay.

Exposure to hydroxyurea or siRNAs against RRM1 and RRM2 activate the Chk1 dependent intra-S checkpoint, as indicated by phosphorylation of Chk1 Ser345 and RPA32 S33 (Figure 2B). Chk1 suppresses DNA damage to maintain cell viability during replication stress [17]. We examined if Chk1 may be required for suppression of DNA damage following siRNA-mediated knockdown of RRM1 and RRM2 by assessing γ-H2AX signal in cells following co-depletion of Chk1 with RRM1 or RRM2. Co-depletion of RRM1 or RRM2 in combination with Chk1 led to significant levels of RPA32 phosphorylation (Figure 3A). In quantitative examinations of DNA damage as measured with γ-H2AX phosphorylation by flow cytometry, single depletion of RRM1, RRM2, and Chk1 for 30 h led to γ-H2AX induction of 9.0%, 23.5%, and 19.3% in transfected cells, compared to 6.6% and 0.1% for luciferase control in the presence or absence of HU (Figure 3B; Table 3). Co-depletion siRNA-mediated knockdowns of RRM1/Chk1 and RRM2/Chk1 resulted in γ-H2AX signal of 34.2% and 81.5%, respectively (Figure 3B; Table 3). Thus, depletion of RRM1/Chk1 led to γ-H2AX signal that was reduced compared to the RRM2/Chk1 and increased to that observed with depletions of RRM1, RRM2 or Chk1 only. Furthermore, specific depletion of RRM1 and RRM2 induces phosphorylation of Chk1 S345 (Figure 3A) suggesting Chk1-dependent checkpoint activation, and combinatorial depletion of RRM1 or RRM2 with Chk1 significantly increases the DNA damage markers (γ-H2AX pS139 and RPA32 pS33).

To verify if Chk1 deficiency could augment RNR effects on cell proliferation and cell death, cells were re-plated following 30 h siRNA-mediated knockdowns of RRM1, RRM2, co-depletion of Chk1 with RRM1 or RRM2 or following 8 hour HU treatment with siRNA-mediated knockdown of Chk1, (positive control) or Luc (negative control). U20S cells harvested at 30 h showed little or no apoptosis compared to Chk1 +HU (positive control) or Luc +HU (negative control) (Figure 3C). Interestingly, 24h post drug release, cells with co-depletion of Chk1 with RRM1 or RRM2 exhibited increased levels of activated caspases similar to Chk1 +HU (Figure 3C) compared to Luc +HU negative control cells. To verify that the apoptosis observed had an effect on cell proliferation, cells were re-plated and allowed to grow for a further seven days as described in Materials and Methods. Figure 3D shows the growth-inhibitory effects following co-depletion of Chk1 with RRM1 or RRM2, consistent with the cell death results. Also, depletion of RRM1 or RRM2 alone showed decreased cell proliferation (Figure 2D, Figure 3D).

Next, we examined if specific depletion of RRM1 and RRM2 and subsequent treatment with a selective Chk1 inhibitor, SCH 900776 (MK8776) [26] would lead to similar induction of γ-H2AX. Cells exposed to SCH 900776 (MK8776) for 2 h following siRNA-mediated knockdown of RRM1 and RRM2 show increased γ-H2AX signal, suppression of DNA synthesis, and accumulation of DNA damage markers (Table 2, Table 3 Figure S2). These data were further corroborated using potent, targeted RRM1 inhibitors in combination with SCH 900776 (MK8776) [29].

Inhibitors of RNR such hydroxurea and gemcitabine are clinically-validated antimetabolites used in cancer treatment and have been reported induce Chk1 S345 and Chk2 T68 phosphorylation [12], [40]. Chk2 T68 was phosphorylated following depletion of RRM2 and to a lesser extent in cells depleted of RRM1 and HU treated cells (Figure 3A). Next, we assessed DNA damage in cells following co-depletion of Chk2 with RRM1 or RRM2. Combinatorial siRNA-mediated knockdown of RRM1/Chk2 and RRM2/Chk2 resulted in γ-H2AX signals of 6.8% and 32.7%, similar to single depletion of RRM1 (9.0%) or RRM2 (23.5%) respectively (Table 3). In contrast to Chk1, Chk2 depletion showed no accumulation of γ-H2AX (Table 3).

To further discern which signalling pathways were essential following HU exposure, cells were co-depleted for Chk1/Chk2 and treated with hydroxyurea for 8 h. Knockdowns were confirmed by Western blot (Figure S3). In quantitative examinations of DNA damage as measured with γ-H2AX phosphorylation by flow cytometry, single depletion of Chk1 and Chk2 led to γ-H2AX signals of 19.3% and 0.2% in transfected cells, compared to 6.6% and 0.1% for luciferase control in the presence or absence of HU (Table 3 Figure S3). Interestingly, single depletions of Chk1 and Chk2 in the presence of HU yielded positive fractions of 60.0% and 9.1% (Table 3; Figure S3). Combinatorial siRNA-mediated knockdown of Chk1/Chk2 yielded γ-H2AX positive fractions of 3.7% compared to single depletions of Chk1 (19.3%) or Chk2 (0.2%) (Table 3; Figure S3). siRNA-mediated knockdown of Chk1 in the presence of HU yielded γ-H2AX positive fractions of 60.0% compared to Chk2 in the presence of HU (9.1%) (Table 3; Figure S3). In contrast to Chk1, Chk2 showed no γ-H2AX increase in the presence of HU. Interestingly, co-depletion of Chk2 with Chk1 appears to suppress the Chk1 γ-H2AX phenotype in the absence or presence of HU. These results suggest that Chk1 and Chk2 play different roles at the DNA replication fork.

### Chk1 interacts with Ribonucleotide reductase

**Figure 4:**
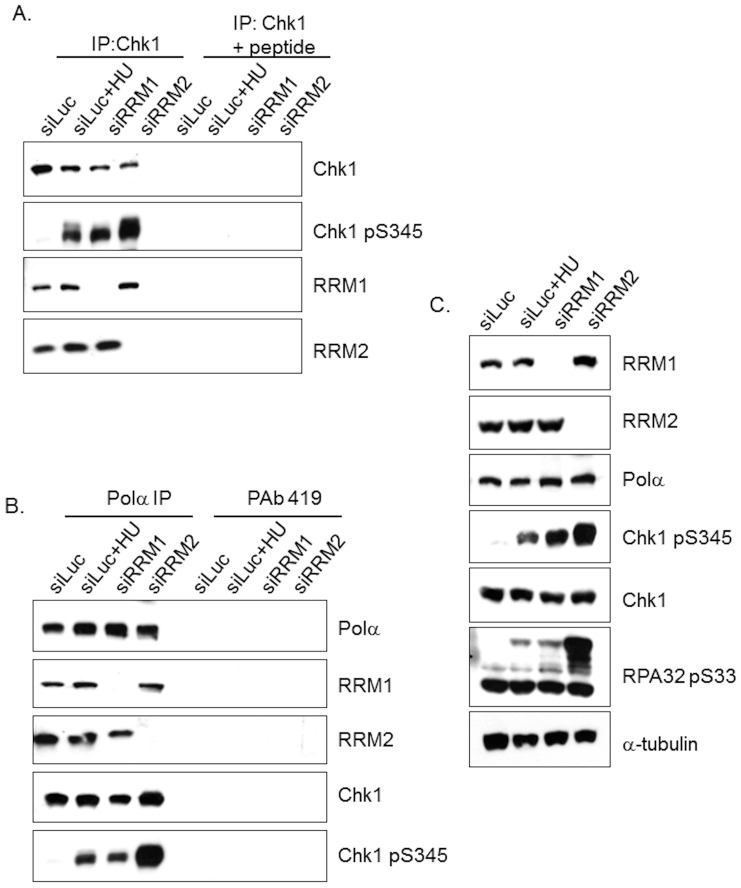
RRM1 and RRM2 subunits of Ribonucleotide reductase interact with Chk1 and Polα. (a) At 30 h after siRNA transfections of the luciferase, RRM1, and RRM2 siRNA duplexes, extracts were prepared for immunoprecipitations. Cells transfected with Luciferase were incubated with 1 mM HU for 30 min. Chk1 was immunoprecipitated from luciferase (positive control), Luciferase + HU, RRM1, and RRM2 depleted cells with Chk1 antibodies (MAb58D7) cross-linked to protein A and were immunoblotted as indicated. Chk1 immunoprecipitations were peptide blocked as a negative control. (b) Polα was immunoprecipitated from luciferase (positive control), luciferase +HU, RRM1, and RRM2 depleted cells depleted cells with Polα antibody (SJK132-20) cross-linked to protein G and Western blots were immunoblotted as indicated. (c) Whole cell extracts were immunoblotted as indicated.

This functional genetic relationship amongst RRM1, RRM2, and Chk1 was reminiscent of that observed between CHK1 and Polα [27] and suggested the possibility of a physical association between RNR, the intra-S checkpoint machinery, and Chk1. Additionally, a proteomics screen to gain insight into Chk1 function identified the RRM1 subunit of Ribonucleotide reductase as associating with Chk1 (Table S1). To validate this potential biochemical interaction, luciferase, RRM1, or RRM2 siRNA duplexes were transfected into U2OS cells and left without further treatment or exposed to HU. Subsequently, Chk1 immune complexes were collected using a specific monoclonal antibody. RRM1 and RRM2 proteins were detected in Chk1 immune complexes and not in immunoprecipitations blocked with Chk1 peptide (Figure 4A). As expected, RRM1 or RRM2 proteins were not detected in Chk1 immunoprecipitations of cells ablated for RRM1 or RRM2, respectively. Significantly, exposure to HU and depletion of RRM1 or RRM2 led to an increase in phosphorylation of Chk1 S345 within total extracts from Chk1 immunoprecipitates. These data suggest that Chk1 associates with RNR in proliferating cells, indicating close connectivity between DNA replication machinery [27] and effectors of replication checkpoint. Western blotting of whole cell extracts validated depletion of RRM1 and RRM2 following siRNA transfection and unchanged levels of Chk1 (Figure 4C). Cells ablated for RRM1 exhibited low levels of DNA damage characterized by high levels of phosphorylation of Chk1 S345 and a functional intra-S checkpoint comparable to luciferase control with HU treatment (Figure 4C). Cells lacking RRM2 showed higher levels of phospho-Chk1 S345 (Figure 4C). These observations are in agreement with prior genetic and functional data in Figure 3 and previously published by Zhou et al. [41] linking Ribonucleotide reductase and Chk1 function.

An important target of antimetabolites is DNA Polα [27]. DNA Polα-Chk1 complex plays a key role during activation of replication checkpoint [27]. Many antimetabolite drugs targeting RNR suppress dNTP levels and inhibit DNA replication [13]–[15]. We next examined whether RNR was detectable in anti-DNA Polα immune complexes. Thus, DNA Polα was immunoprecipitated from U2OS cell extracts prepared from previously depleted of RRM1 or RRM2. Control immunoprecipitations were performed using extracts prepared from cell extracts transfected with luciferase siRNA that are exposed to HU or left untreated. DNA Polα immune complexes contained RRM1, RRM2 and Chk1 protein, in contrast to control IgG immunoprecipitations with Pab419 (Figure 4B). Exposure to HU and depletion of RRM1 or RRM2 led to an increase in phosphorylation of Chk1 S345 in anti-DNA Polα immunoprecipitates. RRM1 or RRM2 was not detected in DNA Polα immunoprecipitations prepared from cells ablated for RRM1 or RRM2. These data suggest that Ribonucleotide reductase interacts with both the DNA Polα complex and the replication checkpoint kinase Chk1.

## Discussion

In this study, we present evidence that Chk1 activation is functionally and genetically linked to Ribonucleotide Reductase (RNR), a finding reinforced by the fact that these critical enzymes in DNA replication checkpoint physically interact in mammalian cells. These functional genomic studies suggest that RNR is a critical mediator of replication checkpoint activation, thus providing new insights into understanding the mechanism of action for RNR and CHK1 in DNA replication and checkpoint.

We observed that cells lacking RRM1 and RRM2 exhibited inhibition of DNA replication as a result of reduced dNTP pools and activation of Chk1 dependent intra-S checkpoint, as indicated by phosphorylation of Chk1 Ser345 and RPA32 S33 and γ-H2AX S139. It was not surprising that inhibition of RRM1 and RRM2 led to reduced cell proliferation (Figure 2D) since both subunits of the RNR are needed to form the active enzyme responsible for maintaining dNTPs levels required for DNA synthesis [1]–[4]. Interestingly, in comparing siRNA-mediated knockdown of the RNR subunits we observed that depletion of RRM2 exhibited stronger phenotypic effects than RRM1 in all experiments (Figure 1, Figure 2; Figure S1). One possibility for these observations is the siRNA-mediated knockdowns for RRM1 and RRM2 may have been sub-optimal due to incomplete transfection. However, no detectable level of protein is seen for either gene, suggesting the knockdown efficiencies are at least beyond detectable thresholds (Figure 2B, Figure 3A). Another possible explanation is that RRM1 and RRM2 may play different roles at the replication checkpoint in addition to regulating dNTP pools. This latter possibility is supported by emerging data suggesting different biological roles for RNR subunits in promoting cancer development [42].

Co-depletion of RRM1 or RRM2 and CHK1 using RNAi leads to dramatic accumulation of DNA damage (γ-H2AX phosphorylation) and induces cell death (Figure 3). Similar γ-H2AX phosphorylation data was observed when using SCH900776 (MK-8776), a functionally selective CHK1 inhibitor currently undergoing clinical trials (Table 3). More recently, these data were further corroborated using novel potent, targeted RRM1 inhibitors in combination with SCH 900776 (MK-8776) [29]. These data suggest that activation of Chk1 is correlated with suppression of catastrophic DNA damage following RNR inhibition using siRNA (Figure 3; Table 3) [41] or inhibitors [17], [26], [29]. These observations are in agreement with genetic and functional data as demonstrated in this study and previously published [29], [41] linking RNR and Chk1 function. Thus, these observations provide a mechanistic understanding for Chk1 and suggest potential therapeutic value of combinations with antimetabolites.

We demonstrate that the RNR interacts with Chk1 and DNA polymerase α in normal cycling cells and when cells are exposed to replication stress (Figure 4; Figure S4). RRM1 was also identified using an affinity purification of Flag-tagged Chk1, coupled with a mass spectrometry proteomic approach to find novel proteins associated with Chk1 (Materials and Methods S1; Table S1). In addition to RRM1 we identified a number of validated proteins that have been shown to be associated with Chk1 such as PRKDC [43], KU86 [43], DDB1 [44], and MCM7 [45] giving us confidence in the screen (Table S1). While a subset of the hits identified in Table 1S has yet to be independently verified by additional methods, others were verified by immunoprecipitaion and Western blot analysis (Figure 4; Figure S4). PRKDC was detected in Chk1 immunoprecipitations in CEM cells which had detectable levels of phosphorylation of Chk1 S345 in response to HU treatment (Figure S4). This is in agreement with localization of PRKDC to DNA breaks in response to replication stress to maintain stability of Chk1 [46]. Surprisingly, the interaction between RRM1 and Chk1 did not require HU treatment in CEM cells (Figure S4) or U2OS cells (Figure 4). Even though previous reports show that both RNR subunits predominantly localized in the cytoplasm [47], [48], this association between RNR and Chk1 or DNA polymerase α is supported by recent studies showing significant detectable signals of both RRM1 and RRM2 proteins in the soluble nuclear and chromatin fractions [49]. Additionally, Alberto et al. [50] have demonstrated that RRM1 localizes to the nucleus using an immunofluorescence-based automated quantitative technique; however, they did not look at RRM2 localization. We speculate that the reason for the Chk1 and RNR interaction is to enable close connectivity between the DNA replication machinery and effectors of replication checkpoint allowing a quick response to replication stress in cells. Further studies will be needed to clarify the significance of the Chk1 and RNR interaction.

In summary, these data provide new insights into the regulation of the replication checkpoint and suggest that RRM1 or RRM2, subunits of RNR are therapeutically relevant oncology drug targets. The goal, therefore, is to discover truly selective RRM1 or RRM2 compounds for use as monotherapy and in combination with CHK1 inhibitors or chemotherapy for the treatment of cancer.

## Supporting Information

Figure S1 **RRM1 and RRM2 depletion using multiple siRNAs lead to phosphorylation of Chk1 and γ-H2AX in U2OS cells.**
(TIF)

Figure S2 **Inhibition of Chk1 with siRNA or clinical compound, SCH900776 (MK8776) with RRM1 or RRM2 shows similar accumulation of DNA damage in U2OS cells.**
(TIF)

Figure S3 **Chk1 depletion not co-depletion of Chk1/Chk2 enhances the accumulation of DNA damage in U2OS cells.**
(TIF)

Figure S4 **Chk1 interacts with RRM1 subunit of Ribonucleotide reductase in CEM cells.**
(TIF)

Table S1 **Identification of Chk1 interacting of potential Chk1 interacting proteins in proteomic screen.**
(DOCX)

Materials and Methods S1 **Generation of Recombinant Adenoviruses, Adenovirus Infections and Immunoprecipitation of Chk1-Flag, SDS-PAGE Separation and In-Gel Digestion, Mass Spectrometry Analysis of Proteins Spots, and Immunoprecipitation.**
(DOCX)
