# Supplementary material for: A Functional Approach Reveals a Genetic and Physical Interaction between Ribonucleotide Reductase and CHK1 in Mammalian Cells
Source: PLoS One. 2014 Nov 6;9(11):e111714. doi: 10.1371/journal.pone.0111714 (PMC4222937; doi:10.1371/journal.pone.0111714)
Supplement: Table S1 — Identification of Chk1 interacting of potential Chk1 interacting proteins in proteomic screen. (DOCX) [file pone.0111714.s005.docx]

**Supporting Information**

**Table S1. Identification of Chk1 interacting of potential Chk1 interacting proteins in proteomic screen.**

| PROTEIN | GENE SYMBOL | NCBI ACCESSION NUMBER | MASCOT SCORE | NUMBER OF  PEPTIDES |
| --- | --- | --- | --- | --- |
| Serine/threonine-protein kinase Chk1 | Chk1 | gi\|4502803 gi\|7531055 gi\|76364111 gi\|76364112 gi\|76364113 | 991 | 101/45 |
| cellular apoptosis susceptibility protein CAS, CSE1 chromosome segregation 1-like (yeast) [Homo sapiens] | CSE1L | gi\|951338 | 42 | 2/1 |
| DNA damage binding protein 1 (DDB p127 subunit) (DDBa) (UV- | DDB1 | gi\|12643730 | 76 | 4/1 |
| ATP-dependent RNA helicase A (DEAH box protein 9) | DDX9 | gi\|2500541 gi\|10435812 | 132 | 4/1 |
| DNA-activated protein kinase, catalytic subunit -human | DNAPKcs | gi\|1362789 gi\|13606056 gi\|13654237 | 28 | 2/1 |
| Heat shock protein 90 | HSP90 | gi\|31615893 gi\|6016264 gi\|74963152 gi\|19859479 gi\|19855062 | 1151 | 68/14 |
| Kinesin-like protein KIF11 (Kinesin-related motor protein Eg5 | KIF11 | gi\|1706622 gi\|114988 | 420 | 21/9 |
| karyopherin beta 1; importin 90; importin beta-1 subunit [Homo sapiens] | KPNB1 | gi\|19923142 | 32 | 2/1 |
| ATP-dependent DNA helicase 2 subunit 1 (ATP-dependent DNA helicase II 70 kDa subunit) | XRCC6  (KU70) | gi\|125729 gi\|4503841 | 96 | 6/1 |
| replication licensing factor MCM7 | MCM7 | gi\|2134885 gi\|20981696 | 31 | 2/1 |
| RRM1 Human mRNA for M1 subunit of ribonucleotide reductase | RRM1 | gi\|4506749 | 35 | 2/1 |
| structural maintenance of chromosomes 4 | SMC4 | gi\|21361252  gi\|30173386 | 25 | 2/1 |
| SWI/SNF complex 60 KDa subunit | SMARCD1 (BAF60A) | gi\|1549243 gi\|4566530 gi\|20070148 gi\|21264348 gi\|31455235 | 28 | 2/1 |
| ATP-dependant DNA helicase II; X-ray repair complementing defective repair in Chinese hamster cells | XRCC5  (KU80) | gi\|10863945 gi\|125731 | 405 | 28/7 |

Table shows a subset of the hits identified by two or more peptides that were not found in the control sample (Supplemental Materials & Methods).

Note: A subset of the hits shown here have yet to be independently verified by additional methods.

Mascot Score = “In Mascot, the ions score for an MS/MS match is based on the calculated probability, P, that the observed match between the experimental data and the database sequence is a random event. The reported score is -10Log(P). The Mascot protein score in the result report from an MS/MS search is derived from the ions scores. For a search that contains a small number of queries, the protein score is the sum of the highest ions score for each distinct sequence.”(www.matrixscience.com/help/interpretation_help.html)

Number of peptides = Total number of peptides identified for each protein hit / Number of peptides for which the ion score is higher than the identity threshold score.
