# Supplementary material for: A Functional Approach Reveals a Genetic and Physical Interaction between Ribonucleotide Reductase and CHK1 in Mammalian Cells
Source: PLoS One. 2014 Nov 6;9(11):e111714. doi: 10.1371/journal.pone.0111714 (PMC4222937; doi:10.1371/journal.pone.0111714)
Supplement: Materials and Methods S1 — Generation of Recombinant Adenoviruses, Adenovirus Infections and Immunoprecipitation of Chk1-Flag, SDS-PAGE Separation and In-Gel Digestion, Mass Spectrometry Analysis of Proteins Spots, and Immunoprecipitation. (DOCX) [file pone.0111714.s006.docx]

**Supporting Information.**

**Materials and Methods S1**

***Generation of Recombinant Adenoviruses***

Full length Chk1 was cloned as Flag-tagged fusion into the adenovirus shuttle vector pAdTrack-CMV (cytomegalovirus). Recombinant adenoviruses were generated and propagated with the pAdEasy system (Agilent technologies) according to the manufacturer’s protocol.

***Adenovirus Infections and Immunoprecipitation of Chk1-Flag***

HEK 293 cells (American Type Culture Collection (ATCC)) were infected for 24 h with recombinant adenoviruses encoding Flag-tagged Chk1 at a multiplicity of infection (MOI) of 10 in 1 ml. Control was uninfected HEK 293 cells. After 24 h of infection, cells were collected by centrifugation at 1000 rpm for 10 min. Supernants were discarded and cell pellets were frozen on dry-ice.

Preparation of cell pellets and immunoprecipitation were all performed according to the manufacturer’s protocol (Sigma) with some modifications. Cell pellets were lysed in Flag lysis buffer (50 mM Tris-HCl pH 7.4, 150 mM NaCl, 1 mM EDTA, 1% Triton-X100, 1:100 dilution of phosphatase inhibitor set I and II, and protease inhibitor cocktail set III (Calbiochem). Lysate were sonicated to solubilize proteins and pre-cleared with Mouse IgG-Agarose. Protein concentrations were determined using the Bio-Rad protein Assay (Bio-Rad). Anti-Flag M2 affinity gel (Sigma) column was prepared according to the manufacturer’s protocol. For immunoprecipitation, protein lysates (5mg) were incubated with the Flag column for 4 hours at 4°C. Proteins bound to Chk1-Flag were eluted with Flag peptide and concentrated using YM-3 Microcon (Millipore) according to the manufacturer’s protocol.

***SDS-PAGE Separation and In-Gel Digestion***

A preparative 4-12% NuPage gel was carried out with an equal amount of each sample and stained with GelCode Coomassie Blue (Pierce, Rockford, IL). For subsequent mass spectrometry analysis, each lane on the gel was manually sliced into equal-sized bands and each band digested with sequencing-grade modified trypsin on a Progest (Genomic Solutions) as described by Shevchenko et al [1].

***Mass Spectrometry Analysis of Proteins Spots***

Mass spectrometry analysis was done on a LCQ Deca Ion Trap (ThermoElectron) with sample introduction with a 48 well Paradigm AS1 autosampler (Michrom Bioresources) and a Paradigm MS4 HPLC system (Michrom Bioresources). The column was self-packed with Vydac C18 resin (5 micron beads, 300Å pores), 10 cm long with a 15 micron tip (New Objectives). The chromatographic separation was done using a linear gradient elution: 8-60% B solvent for 30 minutes (solvent A: 2% acetonitrile, 0.1% formic acid and 0.005% heptaflurobutyric acid, solvent B: 90% acetonitrile, 0.1% formic acid and 0.005% heptaflurobutyric acid).

LC-MS/MS raw files were searched using the Mascot v2 (Matrix Sciences, London, UK) software package against the mouse subset of the National Center for Biotechnology Information (NCBI) non-redundant protein database updated as of July 31, 2006). Search parameters included no restriction on molecular weight and pI, fixed modification on cysteine residues (carbamidomethylation), variable modification on methionine residues (oxidation), a peptide mass tolerance of +/- 1.5 Daltons, a fragment mass tolerance of +/- 0.8 Daltons, and one missed tryptic cleavage. Protein identification was based on at least 2 matching peptides. Protein hits with only one matching peptide were manually reviewed and validated after meeting the following criteria: the MS/MS spectrum must be of good quality with fragment ions clearly above baseline noise and there must be some continuity to the b or y ion series.

***Immunoprecipitation***

Preparation of cell pellets, determination of protein concentrations and CHK1 immunoprecipitation were all performed as previously described [27] in CCRF-CEM (acute lymphoblastic leukemia) cells obtained from ATCC.

**References**

1. Shevchenko A1, Tomas H, Havlis J, Olsen JV, Mann M. (2006) In-gel digestion for mass spectrometric characterization of proteins and proteomes. Nat Protoc 1: 2856-2860.
